# Field testing and psychometric properties of Thai version of the Boston carpal tunnel questionnaire

**DOI:** 10.3389/fneur.2023.1132218

**Published:** 2023-07-18

**Authors:** Pichitchai Atthakomol, Jirawat Nudchapong, Kamonwan Sangseekaew, Worapaka Manosroi, Siam Tongprasert, Tinakon Wongpakaran, Nahathai Wongpakaran

**Affiliations:** ^1^Department of Orthopaedics, Faculty of Medicine, Chiang Mai University, Chiang Mai, Thailand; ^2^English for International Communication Program, International College, Chiang Mai Rajabhat University, Chiang Mai, Thailand; ^3^Division of Endocrinology and Metabolism, Department of Internal Medicine, Faculty of Medicine, Chiang Mai University, Chiang Mai, Thailand; ^4^Department of Rehabilitation Medicine, Faculty of Medicine, Chiang Mai University, Chiang Mai, Thailand; ^5^Department of Psychiatry, Faculty of Medicine, Chiang Mai University, Chiang Mai, Thailand

**Keywords:** Thai, Boston carpal tunnel questionnaire, responsiveness, field testing, validity, reliability

## Abstract

**Introduction:**

The Boston Carpal Tunnel Questionnaire (BCTQ) is a widely recommended patient-reported outcome measure to evaluate symptoms and functions in carpal tunnel syndrome (CTS) patients. We aimed to evaluate the translation and cross-cultural adaptation of the Thai version of the BCTQ (Thai BCTQ) and to investigate the psychometric properties including internal consistency, test-retest reliability, construct validity and responsiveness.

**Methods:**

The Thai BCTQ was field tested with 15 healthy volunteers and 15 CTS patients to evaluate the item-objective congruence of each item. Following that, one hundred and twenty-four CTS patients were included for psychometric evaluation in this study. Internal consistency was assessed using Cronbach’s alpha. Test-retest reliability was examined using the intraclass correlation coefficient (ICC). To evaluate construct validity, Spearman’s rank correlation of the symptom severity scale (Thai BCTQ -S), the functional status scale (Thai BCTQ -F) and the subscales of the Thai MHQ were analyzed. Responsiveness was determined using the standardized response mean (SRM).

**Results:**

Minor modification of the Thai version was made to better explain the term “tingling”. The Thai BCTQ-S, Thai BCTQ-F and Thai BCTQ demonstrated adequate Cronbach’s alpha values (0.91-0.94) and good test-retest reliability (ICC=0.89-0.98). Regarding related dimensions, a strong correlation (*r*=0.67, *P*<0.008) was found between the Thai BCTQ-F and the Function subscale of Thai MHQ as well as between Thai BCTQ-F and the Activities of Daily Living subscale of the Thai MHQ (*r*=0.75, *P*<0.008). In unrelated dimensions, there was a relatively weak correlation between the Thai BCTQ-S and the Aesthetics subscale of the Thai MHQ (r=0.32, *P*=0.0116). The SRM of the Thai BCTQ was 1.46, indicating large responsiveness.

**Discussion:**

The Thai BCTQ has adequate internal consistency in both the symptom and function scales as well as good construct validity and test-retest reliability indicating it is suitable for evaluating Thai CTS patients. This tool also has a high ability to detect clinically significant changes in symptoms and function over time after receiving conservative or surgical treatment.

## Introduction

Carpal tunnel syndrome (CTS) is the most common entrapment neuropathy causing pain, numbness, tingling and weakness of the hand (1, 2). The estimated prevalence of CTS based on diagnostic criteria is up to 14.4% of the general population (3). This condition is most commonly found in females with a peak incidence occurring between the age of 50 and 59 years (4). The suspected cause of CTS is compression of the median nerve at the carpal tunnel resulting from the occupying effect of oedema as well as tendon inflammation, hormonal changes, and manual activity leading to disturbance of the blood flow to the nerve (2). Previous meta-analyzes have demonstrated that diabetes mellitus, hypothyroidism, obesity and pregnancy increase the risk of CTS (5–8).

Currently, patient-reported outcome measures (PROMs) are the standard outcome measurements for evaluating the primary outcomes of clinical practice along with field research based on patients’ perceptions (9–11). Common PROMs used to evaluate clinical outcomes in the general hand and wrist region include the Disabilities of the Arm, Shoulder, and Hand (DASH), the Michigan Hand Outcomes Questionnaire (MHQ) and the Patient-Rated Wrist/Hand Evaluation (PRWHE) (12–17). For assessing disease-specific outcomes, the Boston Carpal Tunnel Questionnaire (BCTQ) is the recommended PROM for evaluating symptoms and function in CTS patients (12, 18). Previous studies have demonstrated that the original BCTQ provides good psychometric properties for assessing clinical outcomes in CTS patients (18, 19). This tool has been translated and cross culturally adapted into many language versions (20–34). Upatham et al. translated the BCTQ into Thai in 2006. However the psychometric property testing including reproducibility, construct validity and responsiveness in that version have not yet been evaluated (35).

The aims of this study were to evaluate the translation and cross-cultural adaptation of the Thai BCTQ and to investigate its psychometric properties including internal consistency, reproducibility, construct validity and responsiveness. We hypothesized that the scales of the Thai BCTQ are internally consistent, reproducible, valid and responsive to clinical change at a level comparable to versions in other languages.

## Materials and methods

This study consisted of 2 processes: reviewing the translation and cross-cultural adaptation of Thai BCTQ and psychometric testing of the Thai BCTQ. The institutional research ethics committee approved this observational study (ORT-2563-07604). Written informed consent was received from all participants.

### The Boston carpal tunnel questionnaire

The BCTQ is a disease-specific PROM for CTS patients. It contains 2 measurement scales: a symptom severity scale (BCTQ-S) and a functional status scale (BCTQ-F). The BCTQ-S uses 11 questions to evaluate the intensity and frequency of pain, numbness, weakness and loss of dexterity on a five-point scale ranging from 1 (no symptoms) to 5 (severe symptoms). The results are interpreted as the average scores of the 11 questions. The BCTQ-F has 8 questions to evaluate the level of difficulty in performing daily tasks, each rated on a five-point scale ranging from 1 (no difficulty) to 5 (cannot do at all due to hand or wrist symptoms). The results are interpreted as the average scores of the 8 questions (18).

### Review of the previous translation and cross-cultural adaptation

A previous Thai version of the BCTQ had been translated and cross-culturally adapted to Thailand following the recommendations of Beaton et al. (35, 36). The process consisted of 5 stages: forward translation, synthesis, back translation and review by an expert committee and field testing (36). However, there had been no field testing of that version of the Thai BCTQ. We field tested the previous Thai BCTQ with 15 healthy volunteers and 15 patients who had been diagnosed with carpal tunnel syndrome based on guidelines for the process of cross-cultural adaptation of self-report measures (36). All participants were native Thai speakers who had the ability to read, write and understand the Thai language fluently. They were invited to evaluate all instructions and questionnaire items for item-objective congruence (IOC) and to provide suggestions. The IOC value is used to evaluate the content validity (37). Briefly, all participants will rate each item based on the degree to which they do or do not measure specific objectives listed by the test developer. The scoring system for each questionnaire item is +1 = clearly measuring, 0 = degree to which it measures the content area is unclear or − 1 = clearly not measuring. The IOC value for each item was calculated using the summation of scores from each participant divided by the number of total participants. The IOC value of 0.5 or more is considered satisfactory (38). If any item had an IOC value less than 0.5, that item was reconstructed and reevaluated until the IOC value was more than 0.5. Finally, the revised Thai BCTQ was translated to back into English and sent to the developers (18) for approval.

### Psychometric testing of the Thai BCTQ

We prospectively enrolled Thai patients having carpal tunnel syndrome at the Orthopedic Outpatient Clinic of the University Hospital between December 2020 and July 2021. Inclusion criteria were patients who had been diagnosed with CTS according to the criteria of practice parameters for carpal tunnel syndrome (39). Any inconclusive diagnoses of CTS were confirmed via electrodiagnosis measurement evaluated by a Rehabilitation Medicine Board-qualified staff member. All patients were between 18 and 70 years old using Thai as their first language. The exclusion criteria were patients who had a musculoskeletal disorder above wrist level, a history of hand or wrist fracture, underlying rheumatoid arthritis, gout, hypothyroidism, active cervical radiculopathy, other concomitant nerve entrapments such as Guyon’s canal or cubital tunnel syndrome, concomitant hand or wrist disorders, e.g., stenosing tenosynovitis or osteoarthritis of the finger or hand at the time of enrollment, pregnancy and an active cerebral disorder or communication problems. We followed the COnsensus-based Standards for the selection of health Measurement Instruments (COSMIN) checklist in conducting this study (40).

Patient demographic data recorded included age, sex, dominant hand, and injured hand. The psychometric properties of Thai BCTQ which were assessed included internal consistency, reproducibility, construct validity and responsiveness.

### Internal consistency

Internal consistency is defined as the degree of interrelatedness among items determining the same outcome. The internal consistency of the Thai BCTQ-S, Thai BCTQ-F and Thai BCTQ was assessed using Cronbach’s alpha. Cronbach’s alpha values range from 0 to 1, with higher scores representing greater interrelatedness between items. Adequate internal consistency is indicated by Cronbach’s alpha values of 0.70 or higher (41).

### Test–retest reliability (reproducibility)

Test–retest reliability is the ability of measurements to obtain the same results in a stable individual (42). The recommended time between the initial and the repeat the measurement is 1 week to avoid recall and to ensure that clinically significant change has not occurred. The Thai BCTQ was administered to CTS patients who had failed conservative treatment and were scheduled for carpal tunnel release; a repeat administration was conducted with the same patients after a 7-day interval. The test–retest reliability of the Thai BCTQ-S, Thai BCTQ-F and Thai BCTQ were measured by intraclass correlation coefficient (ICC), which has a range of from 0 to 1 with ICC values higher than 0.7 indicating good reliability (42).

### Construct validity

Construct validity is a measure of the association between PROMs and the standard instrument which used to evaluate the concepts being measured (42, 43). We used the Thai version of the Michigan Hand Outcomes Questionnaire (Thai MHQ) to assess the construct validity of the concepts being measured (44). We evaluated the correlation between the Thai BCTQ-S, the Thai BCTQ-F and the corresponding subscale of the Thai MHQ using Spearman’s rank correlation coefficient (r). We hypothesized that the same or related subscales should have a high correlation (the Thai BCTQ-S and the Pain subscale of the Thai MHQ, the Thai BCTQ-F and the Function subscale of the Thai MHQ), while unrelated subscales should show a weak correlation (the Thai BCTQ-S and the esthetics subscale of the Thai MHQ, Thai BCTQ-F and the esthetics subscale of the Thai MHQ). The level of correlation was rated as weak (*r* < 0.3), moderate (0.3 ≤ *r* ≤ 0.6), or strong (*r* > 0.6) (44).

### Michigan hand outcomes questionnaire

The Michigan Hand Outcomes Questionnaire (MHQ) is a PROM which is widely used in evaluating hand and wrist regions. It has specific questions in each of 6 subscales including overall hand function (5 items each for the left and right hand), activities of daily living (5 items each for the left and right hand, 7 items for both hands); work performance (5 items); pain (5 items each for the left and right hand); esthetics (4 items each for the left and right hand); and satisfaction with hand function (6 items each for the left and right hand) (12, 13). The MHQ had been translated and cross-culturally adapted to Thai (Thai MHQ) and was shown to have adequate construct validity, reliability and responsiveness for Thai patients (44). Raw scores were converted to scaled scores with a range of 0 to 100. For the Pain subscale, higher scores indicate more pain. In other subscales, higher scores indicate better hand performance (13).

### Responsiveness

Responsiveness is defined as the ability of PROMs to detect clinically significant changes over time (42). We evaluated the responsiveness of the Thai BCTQ-S, Thai BCTQ-F and Thai BCTQ by comparing the scores at baseline and at 12th weeks post-treatment using the standardized response mean (SRM) and effect size (ES) (45). SRM was analyzed as the observed mean change divided by the standard deviation of the observed change while ES was calculated as the observed mean change divided by the standard deviation of the baseline scores. SRM is the preferred value for comparing paired data measurements at different time points for the same patient. SRM and ES values of 0.8, 0.5, and 0.2 were considered to be large, moderate, and small, respectively (45).

### Floor or ceiling effects

Floor or ceiling effects were considered to be present if more than 15% of patients reported the lowest or highest possible scores (46). The responsiveness of patients with the lowest or highest possible score is reduced since changes cannot be evaluated in these patients (42).

### Statistical analysis

For demographic data, categorical variables are reported as frequencies and percentages. Continuous variables are reported as means and standard deviations. Statistical significance was set at *p* < 0.05. In multiple comparisons, the *p*-value was adjusted using Bonferroni’s method. A minimum sample size of 30 participants has been suggested by many statisticians for psychometric analyzes (47).

## Results

### Field testing

Minor cross-cultural adaptations were made to the previous Thai version of the Thai BCTQ-S. During field testing, participants were confused regarding the meaning of the Thai terms for “numbness” and “tingling” which resulted in the IOC of those items being below 0.5. The participants suggested expanding the explanation of the term “tingling.” The developers suggested adding “tingling (paresthesia).” With that change, upon re-evaluation the IOC value of the items which included this term increased to more than 0.5. All other items had an IOC > 0.5, an acceptable level. With this modification, the final Thai BCTQ was approved by the developers (Jeffrey N. Katz and Barry P. Simmons; Supplementary materials S1, S2).

### Psychometric testing

Demographic data are shown in Table 1. One hundred and twenty-four patients were recruited. Most were female and right-handed. The average age was 52 years. Only 12% (15 CTS patients) were defined only on clinical grounds and not by nerve conduction studies. All 124 patients were able to complete the Thai BCTQ without major assistance.

**Table 1:** Demographic data of patients (*n* = 124).

Age (years); mean ± SD	52 ± 12
Female; *n* (%)	110 (89)
Right-hand dominant; *n* (%)	102 (82)
Injured hand; *n* (%)	
Left	21 (17)
Right	43 (35)
Both	
Left more severe	28 (22)
Right more severe	32 (26)
Education level; *n* (%)	
Elementary	41 (33)
Secondary/High School or the equivalent	5 (4)
Diploma	35 (28)
Bachelor’s Degree	28 (23)
Higher than a Bachelor’s degree	15 (12)
Employment status; *n* (%)	
Employed	101 (81)
Unemployed	5 (4)
Retired	18 (15)
Treatment (*n* = 28); *n* (%)	
Surgery	17 (61)
Steroid injection	7 (25)
Splinting	3 (11)
Extracorporeal shockwave therapy	1 (3)

SD: Standard deviation.

### Internal consistency and test–retest reliability

The internal consistency of the Thai BCTQ-S, Thai BCTQ-F and Thai BCTQ was assessed with 124 CTS patients (Table 2). All had adequate internal consistency ranging from 0.91 to 0.94. After excluding patients who did not participate in the follow-up retest after a week and patients who could not complete all the items, test–retest reliability was performed with 49 CTS patients. The results showed the Thai BCTQ-S, Thai BCTQ-F and Thai BCTQ had good reliability with ICC values between 0.89 and 0.98 (Table 3).

**Table 2:** Internal consistency in the Thai BCTQ (*n* = 124).

	Cronbach’s Alpha
BCTQ-S	0.91
BCTQ-F	0.94
BCTQ	0.94

BCTQ: Boston Carpal Tunnel Questionnaire; Symptom severity scale: BCTQ-S; Functional status scale: BCTQ–F.

**Table 3:** Test–retest reliability of the Thai BCTQ (*n* = 49).

Scale	Test	Retest	ICC	95% confidence Interval	
	Mean ± SD	Mean ± SD		Lower bound	Upper bound
BCTQ-S	2.88 ± 0.72	2.82 ± 0.78	0.89	0.81	0.93
BCTQ-F	2.64 ± 0.93	2.59 ± 0.99	0.98	0.97	0.99
BCTQ	2.76 ± 0.72	2.70 ± 0.79	0.96	0.92	0.98

ICC, Intraclass correlation coefficient; BCTQ, Boston Carpal Tunnel Questionnaire; BCTQ-S, Symptom severity scale; BCTQ-F, Functional status scale; SD, Standard deviation.

### Construct validity

After excluding CTS patients who had missing data in the Thai BCTQ or the Thai MHQ, construct validity of the Thai BCTQ was evaluated in CTS 68 patients. The correlations of subscales for the Thai BCTQ and the Thai MHQ were compared (Table 4). For related dimensions, a strong correlation (*r* = 0.61, *p* < 0.008) was found between the Thai BCTQ-S and the Pain subscale of the Thai MHQ, indicating convergent validity. The relationship of the Thai BCTQ-F and the Function subscale of the Thai MHQ had a strong correlation (*r* = 0.67, *p* < 0.008) as was the case with the Thai BCTQ-F and the Activities of daily living subscale of the Thai MHQ (*r* = 0.75, *p* < 0.008). In the case of unrelated dimensions, there were relatively weak correlations between the Thai BCTQ-S and the esthetics subscale of the Thai MHQ (*r* = 0.32, *p* = 0.0116) and between the Thai BCTQ-F and the esthetics subscale of the Thai MHQ (*r* = 0.46, *p* < 0.008) indicating discriminant validity.

**Table 4:** Spearman’s correlation for each subscale among the Thai BCTQ and Thai MHQ (*n* = 68).

MHQ	BCTQ-S	BCTQ-F
Overall hand function	0.52*	0.67*
Activities of daily living	0.50*	0.75*
Work performance	0.47*	0.51*
Pain	0.61*	0.57*
esthetics	0.31	0.46*
Satisfaction with hand function	0.47*	0.55*

* Statistically significant (*p* < 0.008) adjusted for multiple comparison using Bonferroni’s method.

BCTQ, Boston Carpal Tunnel Questionnaire; BCTQ-S, Symptom severity scale; BCTQ-F, Functional status scale; MHQ, Michigan Hand Questionnaire.

### Responsiveness

Twenty-eight patients participated in the followed-up after receiving treatment and completed all items in the Thai BCTQ both before treatment and at follow-up. Eleven patients (39%) received conservative treatment and 17 patients (61%) underwent carpal tunnel release. The SRM and ES of the Thai BCTQ were 1.46 and 1.70, respectively, indicating large responsiveness (Table 5). Subgroup analyzes of SRM and ES of the Thai BCTQ-S, Thai BCTQ-F and Thai BCTQ in CTS patients who had conservative or surgical treatment also showed large responsiveness (Table 5).

**Table 5:** Standardized response mean (SRM) and effect size (ES) of the Thai BCTQ.

		Baseline Mean ± SD	Follow-up Mean ± SD	Mean difference	SD of change	SRM	ES
All treatments (*n* = 28)	BCTQ-S	2.91 ± 0.56	1.85 ± 0.71	1.06	0.83	1.28	1.90
	BCTQ-F	2.73 ± 0.93	1.65 ± 0.79	1.08	0.89	1.21	1.17
	BCTQ	2.82 ± 0.62	1.75 ± 0.70	1.07	0.73	1.46	1.70
Conservative treatment (*n* = 11)	BCTQ-S	2.77 ± 0.41	1.91 ± 0.37	0.85	0.50	1.72	2.08
	BCTQ-F	2.51 ± 0.80	1.48 ± 0.45	1.03	0.71	1.47	1.30
	BCTQ	2.64 ± 0.53	1.69 ± 0.39	0.95	0.51	1.85	1.79
Surgical treatment (*n* = 17)	BCTQ-S	3.01 ± 0.63	1.82 ± 0.87	1.19	0.98	1.21	1.90
	BCTQ-F	2.88 ± 1.00	1.76 ± 0.95	1.11	1.02	1.09	1.11
	BCTQ	2.94 ± 0.67	1.79 ± 0.85	1.15	0.85	1.35	1.72

BCTQ, Boston Carpal Tunnel Questionnaire; BCTQ-S, Symptom severity scale; BCTQ-F, Functional status scale; ES, Effect size; SD, Standard deviation; SRM, Standardized response mean.

### Floor or ceiling effects

No floor or ceiling effects were found in the Thai BCTQ.

## Discussion

The study assessed the psychometric properties of the Thai BCTQ and found that it demonstrated excellent reliability, construct validity and responsiveness. However, some vital points the authors would to address are as follows.

During the process of developing the Thai BCTQ, minor changes were made to clarify the term “tingling,” changing it to “tingling (paresthesia),” since in field testing most participants thought that “numbness” and “tingling” had the same meaning. Some minor modifications were made to the functional status scale in other versions (23, 24, 28). However, there were no major linguistic or cultural discrepancies among the different language versions.

In the evaluation of internal consistency and test–retest reliability, the Thai BCTQ-S, Thai BCTQ-F and Thai BCTQ showed good internal consistency and test–retest reliability with Cronbach’s Alpha and ICC values higher than 0.8. These results are in concordance with other versions (18, 20–34, 48).

In the exploration of construct validity, most publications demonstrated correlation between each scale of the BCTQ and other patient-reported outcome measures (PROMs) such as the Disability of the Arm, Shoulder, and Hand questionnaire (DASH), and the 36-item Short-Form Health Survey (SF-36), Pain Visual Analog Scale, EQ-5D and MHQ (20–22, 24, 25, 27, 29, 34, 48). In this study, we used the MHQ to evaluate the correlation of the subscales to each scale of the BCTQ since the MHQ is more specific in the evaluation of symptoms and function of the hand and wrist regions than DASH, SF-36 and EQ-5D. We found strong correlations between the BCTQ-F and the Function subscale of MHQ, BCTQ-F and Activities of daily living subscale of MHQ as well as the BCTQ-S and the Pain subscale in MHQ. Only weak correlations were found between the BCTQ-S and the esthetics subscale in MHQ and between BCTQ-F and the esthetics subscale in MHQ. These results are comparable to the Polish version of the BCTQ which was evaluated for construct validity using the correlation between the BCTQ and MHQ subscales (25).

The responsiveness of the Thai BCTQ-S, BCTQ-F and BCTQ demonstrated a large standardized response mean (SRM) and effect size (ES). In our cohort, large responsiveness was shown both in CTS patients who had conservative and those who had surgical treatment. Our results are similar to the Swedish, Dutch and Danish versions which showed large SRM and ES in both scales after surgical release (20, 30, 49). Some versions, such as the Korean, Chinese and Japanese, had large SRM and ES in the BCTQ-S and moderate SRM and ES in the BCTQ-F in CTS patients who had surgical release (22, 24, 34). In the comparison of responsiveness of CTS patients who had conservative treatment, another Korean version reported moderate ES in the BCTQ-S and BCTQ-F (48), while our study found a large ES in both. The differences in results might be a consequence of different treatments, e.g., all Korean patients received only steroid injections while CTS patients in our cohort received one of three treatments: steroid injection, splinting or extracorporeal shockwave therapy (48).

To the best of our knowledge, this study was the first to test the psychometric properties of the Thai BCTQ more comprehensively. This study, however, had some limitations. First, although the present study underwent a complete validation process for aspects of validity, reliability and responsiveness compared to other publications (21, 23, 25, 26, 29, 32, 33), a longitudinal study to evaluate the responsiveness of the Thai BCTQ over longer time periods may provide more details of the ability to detect clinical changes. Second, the sample size to evaluate the responsiveness of the Thai BCTQ was quite low. To evaluate the responsiveness of the Thai BCTQ, future research with adequate number of patients should be conducted to confirm our results. Third, the Thai BCTQ should be further evaluated according to item response theory, e.g., the Rasch measurement model, to provide additional information on both persons and item calibration (50).

## Conclusion

The Thai BCTQ has excellent internal consistency in both the symptom and function scales as well as good construct validity and reproducibility for the evaluation of symptoms in Thai CTS patients. This tool also has a high ability to detect clinically significant changes over time following either conservative or surgical treatment. This Thai BCTQ is recommended for use as the standard PROM for clinicians and researchers for the evaluation of symptoms and functions of Thai CTS patients.

## Data availability statement

The raw data supporting the conclusions of this article will be made available by the authors, without undue reservation.

## Ethics statement

The studies involving human participants were reviewed and approved by the Research Ethics Committee of the Faculty of Medicine, Chiang Mai University. The patients/participants provided their written informed consent to participate in this study.

## Author contributions

PA initiated conception and design of the study, performed the collection and acquisition of data, performed the data analysis with interpretation, wrote the manuscript, and responsible for critical revision. JN performed the collection, acquisition of data, and edited the manuscript. KS, ST, TW, and NW participated in data interpretation and manuscript editing. WM performed the data analysis, interpreted the data, and edited the manuscript. This study was supported by Faculty of Medicine, Chiang Mai University.

## Conflict of interest

The authors declare that the research was conducted in the absence of any commercial or financial relationships that could be construed as a potential conflict of interest.

## Publisher’s note

All claims expressed in this article are solely those of the authors and do not necessarily represent those of their affiliated organizations, or those of the publisher, the editors and the reviewers. Any product that may be evaluated in this article, or claim that may be made by its manufacturer, is not guaranteed or endorsed by the publisher.
