# Supplementary material for: Field testing and psychometric properties of Thai version of the Boston carpal tunnel questionnaire
Source: Front Neurol. 2023 Jul 18;14:1132218. doi: 10.3389/fneur.2023.1132218 (PMC10392831; doi:10.3389/fneur.2023.1132218)
Supplement: Supplementary file 1 [file Data_Sheet_1.docx]

**คำชี้แจง** คำถามต่อไปนี้หมายถึงอาการของท่านในช่วง 2 สัปดาห์ที่ผ่านมา โปรดวงกลมหรือกากบาทคำตอบที่ตรงกับอาการของท่านมากที่สุด

**หมายเหตุ** : ถ้ามีอาการ 2 ข้าง เลือกข้างที่มีอาการมากที่สุดในการตอบคำถาม

1. อาการปวดบริเวณมือ หรือ ข้อมือ ในช่วงเวลากลางคืนของท่านมีมากน้อยเพียงใด

1. ไม่มีอาการปวดบริเวณมือ หรือข้อมือ เวลากลางคืนเลย

2. มีอาการปวด เพียงเล็กน้อย

3. มีอาการปวด ปานกลาง

4. มีอาการปวด รุนแรง

5. มีอาการปวด รุนแรงมาก

2. ตลอดเวลา 2 สัปดาห์ ท่านมีอาการปวดบริเวณมือ หรือ ข้อมือ จนทำให้ต้องตื่นนอนกลางคืนเพราะอาการปวดบ่อยเพียงใด

1. ไม่เคยตื่นนอนกลางคืนเพราะอาการปวดเลย

2. ตื่นนอนเพราะอาการปวด 1 ครั้ง

3. ตื่นนอนเพราะอาการปวด 2 – 3 ครั้ง

4. ตื่นนอนเพราะอาการปวด 4 – 5 ครั้ง

5. ตื่นนอนเพราะอาการปวด มากกว่า 5 ครั้ง

3. ท่านมีอาการปวดบริเวณมือ หรือ ข้อมือ ในช่วงเวลากลางวันหรือไม่

1. ไม่เคยมีอาการปวดเวลากลางวันเลย

2. มีอาการปวด เพียงเล็กน้อย

3. มีอาการปวด ปานกลาง

4. มีอาการปวด รุนแรง

5. มีอาการปวด รุนแรงมาก

4. ท่านมีอาการปวดบริเวณมือ หรือ ข้อมือ ในช่วงเวลากลางวัน บ่อยเพียงใด

1. ไม่เคยมีอาการปวดช่วงเวลากลางวันเลย

2. มีอาการปวด วันละ 1 – 2 ครั้ง

3. มีอาการปวด วันละ 3 – 5 ครั้ง

4. มีอาการปวด วันละ มากกว่า 5 ครั้ง

5. มีอาการปวดตลอดทั้งวัน

5. หากท่านมีอาการปวดในช่วงกลางวัน ในแต่ละครั้งที่มีอาการปวด อาการนั้นคงอยู่นานเท่าใด

1. ไม่เคยมีอาการปวดช่วงเวลากลางวันเลย

2. ระยะเวลาที่ปวด แต่ละครั้งน้อยกว่า 10 นาทีต่อครั้ง

3. ระยะเวลาที่ปวด แต่ละครั้งอยู่ในช่วง 10 – 60 นาทีต่อครั้ง

4. ระยะเวลาที่ปวด แต่ละครั้งมากกว่า 60 นาทีต่อครั้ง

5. อาการที่ปวด คงที่ตลอดเวลา

6. ท่านมีอาการชา(ความรู้สึกลดลง) บริเวณมือหรือไม่

1. ไม่มีอาการชาบริเวณมือเลย

2. มีอาการชา เพียงเล็กน้อย

3. มีอาการชา ปานกลาง

4. มีอาการชา รุนแรง

5. มีอาการชา รุนแรงมาก

7. ท่านมีอาการอ่อนแรงบริเวณมือ หรือ ข้อมือหรือไม่

1.ไม่มีอาการอ่อนแรงของมือ หรือ ข้อมือเลย

2. มีอาการอ่อนแรง เพียงเล็กน้อย

3. มีอาการอ่อนแรง ปานกลาง

4. มีอาการอ่อนแรง มาก

5. มีอาการอ่อนแรง มากที่สุด

8. ท่านมีอาการเหน็บชา(รู้สึกซ่าคล้ายไฟช็อต) บริเวณมือหรือไม่

1. ไม่มีอาการเหน็บชาบริเวณมือเลย

2. มีอาการเหน็บชา เพียงเล็กน้อย

3. มีอาการเหน็บชา ปานกลาง

4. มีอาการเหน็บชา รุนแรง

5. มีอาการเหน็บชา รุนแรงมาก

9. ท่านมีอาการชา(ความรู้สึกลดลง) หรือเหน็บชา(รู้สึกซ่าคล้ายไฟช๊อต) บริเวณมือ ในช่วงกลางคืนหรือไม่

1. ไม่มีอาการชาหรือเหน็บชาบริเวณมือในช่วงกลางคืนเลย

2. มีอาการชาหรือเหน็บชาในช่วงกลางคืน เพียงเล็กน้อย

3. มีอาการชาหรือเหน็บชาในช่วงกลางคืน ปานกลาง

4. มีอาการชาหรือเหน็บชาในช่วงกลางคืน รุนแรง

5. มีอาการชาหรือเหน็บชาในช่วงกลางคืน รุนแรงมาก

10. ในช่วง 2 สัปดาห์ที่ผ่านมา ท่านมีอาการชา(ความรู้สึกลดลง) หรือ เหน็บชา(รู้สึกซ่าคล้ายไฟช็อต) ที่มือ ในเวลากลางคืนอย่างรุนแรง จนทำให้ท่านต้องตื่นนอนเวลากลางคืน ประมาณกี่ครั้ง

1. ไม่เคยมีอาการจนต้องทำให้ต้องตื่นนอนเวลากลางคืนเลย

2. 1 ครั้ง

3. 2 – 3 ครั้ง

4. 4 – 5 ครั้ง

5. มากกว่า 5 ครั้ง

11. ท่านรู้สึกมีความยากลำบาก ในการกำวัตถุสิ่งของ และใช้มือจับวัตถุขนาดเล็กๆ เช่น กุญแจ หรือ ปากกา หรือไม่

1. ไม่มีความยากลำบากเลย

2. มีความยากลำบาก เพียงเล็กน้อย

3. มีความยากลำบาก ปานกลาง

4. มีความยากลำบาก มาก

5. มีความยากลำบาก มากที่สุด

**คำชี้แจง** โปรดประเมินความสามารถของการใช้มือข้างที่มีอาการ ในช่วงเวลา 2 สัปดาห์ที่ผ่านมา ท่านมีความยากลำบากในการใช้มือทำกิจกรรมดังต่อไปนี้หรือไม่ โดยให้ท่านวงกลมหรือกากบาทลงบนคำตอบที่ตรงกับความสามารถของท่านมากที่สุด

**หมายเหตุ** : ถ้ามีอาการ 2 ข้าง เลือกข้างที่มีอาการมากที่สุดในการตอบคำถาม

| กิจกรรม | ไม่มีความยากลำบากเลย | ยากลำบาก  เล็กน้อย | ยากลำบาก  ปานกลาง | ยากลำบาก  มาก | ไม่สามารถใช้มือ  ทำกิจกรรมนี้ได้เลย |
| --- | --- | --- | --- | --- | --- |
| เขียนหนังสือ | 1 | 2 | 3 | 4 | 5 |
| ติดกระดุมเสื้อ | 1 | 2 | 3 | 4 | 5 |
| ถือหนังสือขณะอ่าน | 1 | 2 | 3 | 4 | 5 |
| ถือโทรศัพท์ | 1 | 2 | 3 | 4 | 5 |
| เปิดขวด, กระปุก | 1 | 2 | 3 | 4 | 5 |
| ทำงานบ้าน | 1 | 2 | 3 | 4 | 5 |
| ถือถุงหิ้ว | 1 | 2 | 3 | 4 | 5 |
| อาบน้ำแต่งตัว | 1 | 2 | 3 | 4 | 5 |
