# Supplementary material for: Field testing and psychometric properties of Thai version of the Boston carpal tunnel questionnaire
Source: Front Neurol. 2023 Jul 18;14:1132218. doi: 10.3389/fneur.2023.1132218 (PMC10392831; doi:10.3389/fneur.2023.1132218)
Supplement: Supplementary file 2 [file Data_Sheet_2.docx]

**Instruction:** These questions refer to your symptoms in the past two weeks. Please draw a circle or mark with a cross at the answer which is the most related to your symptom.

**Remarks:** In case that it happened with both sides of your hands or wrists, choose the one with the most severe symptom to answer the questions.

1. How painful was your hand or wrist pain during nighttime?
2. I never felt pain during nighttime.
3. I felt slight pain.
4. I felt moderate pain.
5. I felt severe pain.
6. I felt unbearable pain.
7. In the past two weeks, how many times did you feel so painful that it awoke you in the middle of the night?
8. I was never awoken because of pain.
9. I was awoken because of pain one time.
10. I was awoken because of pain two to three times.
11. I was awoken because of pain four to five times.
12. I was awoken because of pain more than five times.
13. Did you have hand or wrist pain during daytime?
14. I never felt pain during daytime.
15. I felt slight pain.
16. I felt moderate pain.
17. I felt severe pain.
18. I felt unbearable pain.
19. How many times did you feel pain during daytime?
20. I never felt pain during daytime
21. I felt pain one to two times.
22. I felt pain three to five times.
23. I felt pain more than five times.
24. I felt pain all day.

5. In case that you felt pain during daytime, how long did it take for each time?

1. I never felt pain during daytime.
2. It was less than 10 minutes.
3. It was from 10 to 60 minutes.
4. It was more than 60 minutes.
5. The pain was constant.

6. Did you feel numb (less sensation) in your hand?

1. I never felt numb in my hands.

2. I felt slightly numb.

3. I felt moderately numb.

4. I felt severely numb.

5. I felt unbearably numb.

7. Did you have hands or wrists weakness?

1. I never had hands or wrists weakness.

2. I had slight hands or wrists weakness.

3. I had moderate hands or wrists weakness.

4. I had severe hands or wrists weakness.

5. I had unbearable hands or wrists weakness.

8. Did you feel tingling (paresthesia) in your hands?

1. I never felt tingling (paresthesia) in my hands.

2. I felt slight tingling (paresthesia).

3. I felt moderate tingling (paresthesia).

4. I felt severe tingling (paresthesia).

5. I felt unbearable tingling (paresthesia).

9. Did you feel numb (less sensation) or tingling (paresthesia) in your hands during nighttime?

1. I never feel numb nor tingling in my hands during nighttime.

2. I felt slightly numb or tingling in my hands during nighttime.

3. I felt moderately numb or tingling in my hands during nighttime.

4. I felt severely numb or tingling in my hands during nighttime.

5. I felt unbearably numb or tingling in my hands during nighttime.

10. In the past two weeks, how many times did you feel numb (less sensation) or tingling (paresthesia) in your hands during nighttime that awoke you in the middle of the night?

1. I never had any symptoms that awoke me in the middle of the night.

2. One time

3. Two to three times

4. Four to five times

5. More than five times

11. Did you find any difficulties in grasping objects or holding small objects such as a key or a pen?

1. I found no difficulties.

2. I found slightly difficulties.

3. I found moderately difficulties.

4. I found severely difficulties.

5. I found unbearably difficulties.

**Instruction:** Please evaluate your hand with symptoms’ ability in the past two weeks. Did you find any difficulties in doing following activities? Please draw a circle or mark with a cross at the answer which is the most related to your ability.

**Remarks:** In case that it happened with both sides of your hands or wrists, choose the one with the most severe symptom to answer the questions.

| Activities | No difficulties | Slight difficulties | Moderate difficulties | Severe difficulties | Unbearable difficulties |
| --- | --- | --- | --- | --- | --- |
| Writing | 1 | 2 | 3 | 4 | 5 |
| Buttoning a shirt | 1 | 2 | 3 | 4 | 5 |
| Holding a book while reading | 1 | 2 | 3 | 4 | 5 |
| Holding a phone | 1 | 2 | 3 | 4 | 5 |
| Opening a bottle or a jar | 1 | 2 | 3 | 4 | 5 |
| Doing housework | 1 | 2 | 3 | 4 | 5 |
| Holding shopping bags | 1 | 2 | 3 | 4 | 5 |
| Taking shower and getting dressed | 1 | 2 | 3 | 4 | 5 |
